# Exogenous IFN-beta regulates the RANKL-c-Fos-IFN-beta signaling pathway in the collagen antibody-induced arthritis model

**DOI:** 10.1186/s12967-014-0330-y

**Published:** 2014-12-10

**Authors:** Rong Zhao, Ni-Nan Chen, Xiao-Wei Zhou, Ping Miao, Chao-Ying Hu, Liu Qian, Qi-Wen Yu, Ji-Ying Zhang, Hong Nie, Xue-hua Chen, Pu Li, Rong Xu, Lian-Bo Xiao, Xin Zhang, Jian-Ren Liu, Dong-Qing Zhang

**Affiliations:** Department of Neurology, Shanghai Ninth People’s Hospital, Shanghai Jiao Tong University School of Medicine, Shanghai, 200011 China; Shanghai Institute of Immunology, Shanghai Jiao Tong University School of Medicine, Shanghai, 200025 China; Central laboratory, Shanghai Xuhui Central Hospital, Shanghai, 200031 China; Shanghai Ruijin Hospital, Shanghai Jiao Tong University School of Medicine, Shanghai, 200025 China; Department of Central laboratory, Shanghai Guanghua Hospital of Integrated Traditional Chinese and Western Medicine, Shanghai, 200052 China

**Keywords:** Rheumatoid arthritis, Interferon-β, Collagen II antibody-induced arthritis, Receptor activator of nuclear factor κB ligand, c-Fos

## Abstract

**Background:**

Although a variety of drugs have been used to treat the symptoms of rheumatoid arthritis (RA), none of them are able to cure the disease. Interferon β (IFN-β) has pleiotropic effects on RA, but whether it can be used to treat RA remains globally controversial. Thus, in this study we tested the effects of IFN-β on RA patients and on collagen antibody-induced arthritis (CAIA) model mice.

**Methods:**

The cytokine and auto-antibody expression profiles in the serum and synovial fluid (SF) from RA patients were assessed using enzyme-linked immunosorbent assay (ELISA) and compared with the results from osteoarthritis (OA) patients. Exogenous IFN-β was administered to RA patients and CAIA model mice, and the therapeutic effects were evaluated. Endogenous IFN-β expression in the joint bones of CAIA model mice was evaluated by quantitative real-time PCR (qRT-PCR). The effects of exogenous IFN-β on CAIA model mice were assessed using a clinical scoring system, hematoxylin eosin and safranin-O with fast green counterstain histology, molybdenum target X-ray, and tartrate-resistant acid phosphatase (TRAP) staining. The RANKL-RANK signaling pathway was analyzed using qRT-PCR. The RAW 264.7 cell line was differentiated into osteoclasts with RANKL stimulation and then treated with exogenous IFN-β.

**Results:**

The expression of inflammatory cytokines (IFN-γ, IL-17, MMP-3, and RANKL) and auto-antibodies (CII antibodies, RF-IgM, and anti-CCP/GPI) were significantly higher in RA compared with OA patients. After IFN-β intervention, some clinical symptoms in RA patients were partially alleviated, and the expression of IFN-γ, IL-17, MMP-3, and OPG) returned to normal levels. In the CAIA model, the expression of endogenous IFN-β in the joint bones was decreased. After IFN-β administration, the arthritis scores were decreased; synovial inflammation, cartilage, and bone destruction were clearly attenuated; and the expression of c-Fos and NFATc1 were reduced, while RANKL and TRAF6 expression was unchanged. In addition, exogenous IFN-β directly inhibited RANKL-induced osteoclastogenesis.

**Conclusions:**

Exogenous IFN-β administration immunomodulates CAIA, may reduce joint inflammation and, perhaps more importantly, bone destruction by inhibiting the RANKL-c-Fos signaling pathway. Exogenous IFN-β intervention should be selectively used on RA patients because it may only be useful for RA patients with low endogenous IFN-β expression.

## Background

Rheumatoid arthritis (RA) is an autoimmune disease that is characterized by chronic inflammation of the synovial joints, with subsequent progressive erosion and destruction of the articular tissues [1,2]. RA affects around 1% of the population and is associated with significant morbidity and mortality [3]. Although a variety of drugs have been used to treat the symptoms, none of them are able to cure the disease. Disease-modifying anti-rheumatic drugs, such as methotrexate, are one of the most common types of treatments. Other efficient anti-rheumatic drugs have recently been developed, including biological response modifiers such as tumor necrosis factor (TNF)-α blockers [4,5]. However, the clinical use of these therapies is limited because of their adverse effects and high cost. Therefore, developing novel therapies is crucial for RA patients.

For many years, IFN-β was assumed to be beneficial for the treatment of a variety of immune mediated diseases. When administered to multiple sclerosis (MS) patients, IFN-β reduces the relapse rate, decreases the disease activity on magnetic resonance imaging (MRI) scans, and delays the progression of disability [6,7]. IFN-β has clear anti-inflammatory properties, and, moreover, it plays an important role in maintaining bone homeostasis by inhibiting osteoclastogenesis. IFN-β may be an effective treatment for RA [8,9]. Previous studies have shown that IFN-β therapy is effective in murine models of arthritis; however, systemic IFN-β treatment results in only minimal improvement in RA [8,10,11]. In order to assess the effects of IFN-β on RA, exogenous IFN-β was administered to RA patients and collagen antibody-induced arthritis (CAIA) model mice in this study. The mechanism of beneficial IFN-β effects on CAIA was also evaluated, specifically the signaling pathway effected during osteoclastogenesis.

## Methods

### Patients

Patients with RA or osteoarthritis (OA) were enrolled in this study from 2008 to 2013 in the Shanghai Guanghua Hospital of Integrated Traditional Chinese and Western Medicine (Shanghai, China). All the RA patients fulfilled the 1987 revised criteria of the American College of Rheumatology (formerly the American Rheumatism Association) [12,13], and the OA patients fulfilled the American College of Rheumatology criteria [14]. Informed consent was obtained from each participant, and the experimental protocol was approved by the hospital’s Human Research Ethics Committee.

### Exogenous IFN-β intervention in RA patients

Twenty RA patients were selected for an immune interference study with exogenous IFN-β (Rebif®, Merck Serono, Darmstadt, Germany) administered as in the MS and phase I clinical trials for RA patients [7,12]. A clinical assessment was performed by evaluating the duration of morning stiffness (min), the number of painful joints and swollen joints, and the degree of pain (by Visual Analog Scale [VAS]) in RA patients both before and after exogenous IFN-β administration.

### Enzyme-linked immunosorbent assay (ELISA)

Peripheral blood samples from 22 RA and 13 OA patients, as well as synovial fluid (SF) from 21 RA and 5 OA patients, were collected under aseptic conditions. The levels of inflammatory cytokines interleukin-17 (IL-17), interferon γ (IFN-γ), tissue inhibitor of metalloproteinases 1 (TIMP-1), matrix metalloproteinase 3 (MMP-3), osteoprotegerin (OPG), and receptor activator of nuclear factor κB (RANKL), as well as CII antibody, rheumatoid factor-IgM (RF-IgM), anti-cyclic citrullinated peptide antibody (CCP), and glucose-6-phosphate isomerase antibodies (GPI) were detected using Quantikine ELISA kits (R&D Systems, Minneapolis, MN, USA) according to the manufacturer’s instructions. Thresholds of CII IgA/CII IgG >2.2 U/mL, CII IgM >2.4 U/mL, RF-IgM >20 U/mL, GPI >2.0 mg/L, and anti-CCP >5 U/mL were used to identify positive samples according to the standards of the clinical laboratory of Shanghai Guanghua Hospital of Integrated Traditional Chinese and Western Medicine.

### Animals

BALB/c mice (20–23 g, 8–10 weeks) were purchased from the Chinese Academy of Sciences, Shanghai Laboratory Animal Center and housed following institutional guidelines. Experiments were conducted according to the guidelines of the Ethics Committee of Laboratory Animals Welfare of Shanghai Jiao Tong University School of Medicine.

### Induction of CAIA and establishment of the treatment protocol

To induce the CAIA model, BALB/c mice were injected with 2 mg of collagen antibody cocktail (Chondrex, Redmond, WA, USA) intravenously on Day 1, and were then treated with 25 μg of lipopolysaccharide (LPS) intraperitoneally on Day 4. All the mice were monitored daily for arthritis. Each paw was scored for clinical signs of arthritis as follows: normal (0); erythema and edema in only one digit (0.5); erythema and mild edema of the footpad, ankle, or two to five digits (1); erythema and moderate edema of two joints (footpad, ankle, or two to five digits) (2); erythema and severe edema of the entire paw (3); reduced swelling and deformation leading to incapacitation of the limb (4). Each mouse arthritic score was obtained by summing the scores recorded for each paw. The clinical evaluations were performed by two blinded investigators, and the mean of both scores was calculated [15]. On Day 4, after LPS injection, the intervention group CAIA model mice (n = 9) received 10,000 IU of exogenous mouse IFN-β (PBL interferon source, Piscataway, NJ, USA) every day by intraperitoneal injection for 4 days, while the control group (non-intervention group) CAIA model mice (n = 9) were similarly treated with sterile saline.

### Molybdenum X-ray imaging

Prior to histology, molybdenum X-ray radiographs (Adobe Systems, Munich, Germany) of the knees and paws of each mouse were taken on day 12 after induction of arthritis. The limbs were extended to prevent joint buckling, and the bone mineral density was assessed.

### Histology

At day 12 after induction of arthritis, the knees and paws were harvested and fixed in 4% paraformaldehyde, decalcified, and embedded in paraffin. Serial sections of the knees and paws were stained with hematoxylin and eosin (H&E, Sakura Finetek, Tokyo, Japan) or safranin-O with fast green counterstain. Inflammation and joint damage were scored on a scale of 0 (no inflammation) to 3 (severe inflammation) depending on the number of inflammatory cells. Cartilage destruction was scored on a scale of 0 (no loss) to 3 (complete loss of the articular cartilage). Scoring was performed by two blinded investigators, and the mean of both scores was calculated.

### Quantitative real-time polymerase chain reaction (qRT-PCR)

The hind paws and joint bones of the CAIA model mice were pulverized in liquid nitrogen, and the total RNA was extracted using TRIzol® reagent (Invitrogen, Carlsbad, CA, USA). One μg of the total RNA was reverse transcribed using a reverse transcription kit (Promega, Madison, WI, USA). Quantitative real-time PCR (qRT-PCR) was performed with duplicate samples on the ABI7500 system (Applied Biosystems, Darmstadt, Germany) under the following conditions: 2 min of polymerase activation at 95°C followed by 45 cycles of 10 sec denaturation at 95°C and 30 sec annealing and extension at 60°C. The detection threshold was set to the log linear range of the amplification curve and kept constant (0.05) for all data analysis. Threshold cycle (*C*_*T*_) of each target product was determined and set in relation to the amplification plot of β-actin. Differences in the *C*_*T*_ values (Δ*C*_*T*_) between each gene and β-actin were used to calculate the relative expression (relative expression = 2^−(*CT* of target genes−^^*CT* of β-actin)^ =2^−Δ*CT*^). The mouse PCR primers (Sangon Biotech, Shanghai, China) used for RT-PCR were as follows: for IFN-β, sense: 5′-CGTTCCTGCTGTGCTTCTC-3′ and anti-sense: 5′-TGTAACTCTTCTCCATCTGTGAC-3′; TIMP-1, sense: 5′-GCCGCCATCATCGCAGAT-3′ and anti-sense: 5′- CCTTATGACCAGGTCCGAGTTG-3′; MMP-3, sense: 5′- AAGAGATCCAAGGAAGGCATCCT-3′ and anti-sense: 5′- GGTTCTGCCATAGCACATGCT-3′; TRAP, sense: 5′-AAATCACTCTTCAAGACCAG-3′ and anti-sense: 5′-TTATTGAACAGCAGTGACAG-3′; RANKL, sense: 5′-TGCCGCTACCGCAAGACAGA-3′ and anti-sense: 5′-GCAGGCTTACGTTGGCTCCC-3; TRAF-6, sense: 5′-GCTCAAACGGACCATTCGGA-3′ and anti-sense: 5′-GGGATTGTGGGTCGCTGAAA-3′; c-Fos, sense: 5′-CCCTTTGATGACTTCTTGTTTCCG-3′ and anti-sense: 5′-AATTGCTGTGCAGAGGCTCCC-3′; NFATc1, sense: 5′-TCTCGAAAGACAGCACTGGAGCAT-3′ and anti-sense: 5′-ACGGGATCTCCAGGAATTTGGTGT-3′; β-actin, sense: 5′-CTGTCCCTGTATGCCTCTG-3′ and anti-sense: 5′-ATGTCACGCACGATTTCC-3′.

### Cell culture and differentiation

The murine macrophage cell line RAW 264.7 (generously provided by Dr. J. Luo, East China Normal University) was plated in 24-well plates (10,000 cells per well) containing α-minimum essential medium (α-MEM) supplemented with 10% fetal calf serum (FCS). The cells were stimulated with 50 ng/mL RANKL (R&D Systems) with or without exogenous mouse IFN-β (50 IU/mL) for 4 days. All cells were cultured in a 5% CO_2_/95% air incubator. The culture medium was replaced with fresh medium every day.

### Tartrate-resistant acid phosphatase (TRAP) staining

The paraffin-embedded sections of the joint bones of the CAIA model mice and RANKL-induced osteoclastogenesis on the fourth day after induction were gently washed twice with pre-warmed, double-distilled water (37°C), fixed with stationary liquid for 20 sec, and stained with tartrate-resistant acid phosphatase (TRAP, Sigma, St. Louis, MO, USA) for 60 min at 37°C. The TRAP-stained cells were then gently washed, counterstained in the dark with hematoxylin or 100 μL/well of 300 nM diamidino-2-phenylindole (DAPI ) in phosphate buffer solution (PBS) containing 0.1% Triton X-100 at room temperature for 15 min, and examined with a ZEISS Vert.A1 microscope (Carl Zeiss, Oberkochen, Germany). TRAP-positive cells appeared dark red, and TRAP-positive multi-nucleated cells containing three or more nuclei were counted as osteoclasts. Osteoclasts were quantified by imaging five fields of view under 200× magnification and directly counting the number of TRAP-positive cells [16]. All experiments were carried out in triplicate at least 3 times.

### Statistical analyses

Statistical analyses were performed in Prism (GraphPad Software, La Jolla, CA, USA). Values are presented as mean ± standard deviation. Unpaired two-tailed Student’s *t-*tests were used for parametric outcomes to compare groups. The Kruskal–Wallis test for several group means followed by the Mann–Whitney U test for comparison of two groups [17] were also used. *P*-values <0.05 were considered statistically significant. *: *P* <0.05, **: *P* <0.01.

## Results

### Differences in the level of inflammatory factors and auto-antibodies between RA and OA patients

**Figure 1:**
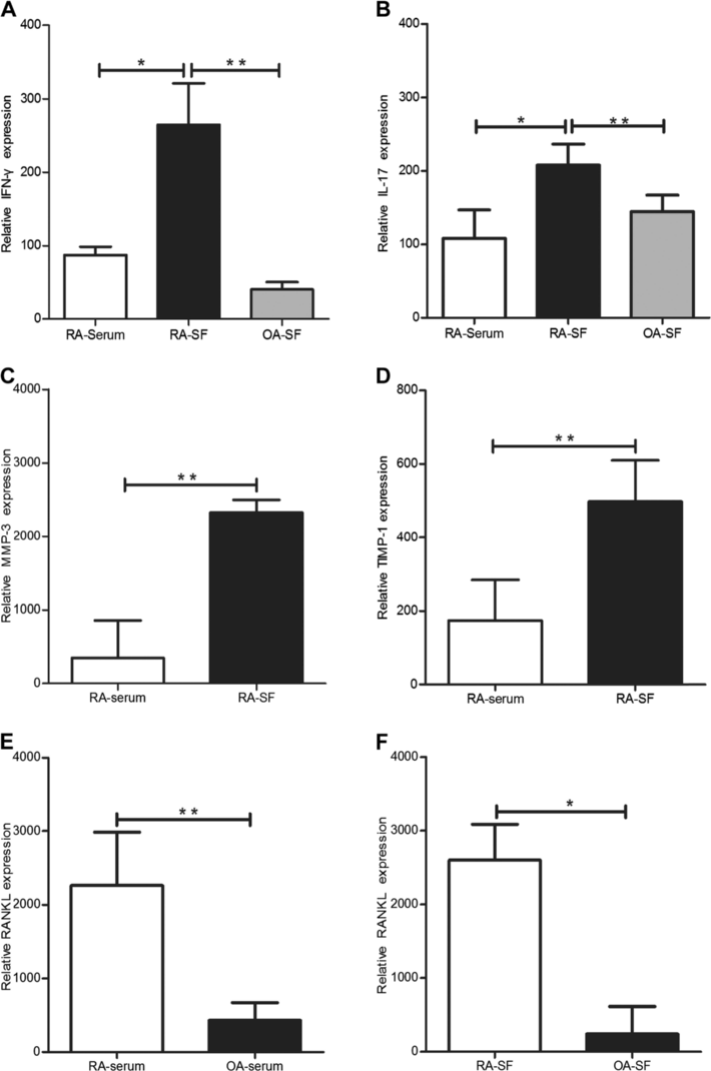
**The expression of inflammatory factors in the serum and SF of RA patients.** The levels of IFN-γ **(A)** and IL-17 **(B)** in the RA SF were compared with that in RA serum and OA SF. The levels of MMP-3 **(C)** and TIMP-1 **(D)** in the serum and SF of RA patients were assessed. The levels of RANKL in RA serum **(E)** and SF **(F)** were compared with those in OA serum and SF. *: *P* <0.05, **: *P* <0.01.

The level of inflammatory factors assessed by ELISA in RA serum and SF were compared with those in OA serum and SF. IFN-γ and IL-17 were significantly higher in RA SF than RA serum (*P* <0.05), and also higher than that in OA SF (Figure 1A,B) (*P* <0.01). The levels of MMP-3 and TIMP-1 were higher in RA SF than RA serum (Figure 1C,D) (*P* <0.01). The levels of RANKL were significantly higher in RA serum and SF compared with that in OA serum and SF (Figure 1E,F) (*P* <0.01, *P* <0.05). For auto-antibodies, the positive rate of IgG was higher in RA SF than in OA SF (Table 1). The positive rates of RF-IgM, anti- CCP, and GPI in RA serum were higher than those in OA serum (Table 2).

**Table 1:** **The fraction of samples positive for CII IgA, IgG, and IgM in RA and OA serum and SF**

**Group**	**CII antibody**		
	**IgG(+/-)**	**IgA(+/-)**	**IgM(+/-)**
RA Serum (n = 22)	6/16	4/18	3/19
OA Serum (n = 6)	2/4	2/4	2/4
RA SF (n = 21)	15/6*	8/13	12/9
OA SF (n = 5)	0/5*	0/5	1/4

CII: collagen II; RA: rheumatoid arthritis; OA: osteoarthritis; SF: synovial fluid. *: *P* <0.05.

**Table 2:** **The fraction of samples positive for RF-IgM, Anti-CCP, and GPI in RA and OA serum**

**Group**	**RF-IgM(+/-)**	**Anti-CCP(+/-)**	**GPI(+/-)**
RA serum (n = 22)	17/5*	15/7**	14/8**
OA serum (n = 13)	4/9*	0/13**	2/11**

RF-IgM: rheumatoid factor-IgM; Anti-CCP: anti-cyclic citrullinated peptide antibody; GPI: glucose-6-phosphate isomerase antibodies; RA: rheumatoid arthritis; OA: osteoarthritis. *: *P* <0.05, **: *P* <0.01.

### Cytokine levels were altered by IFN-β administration

**Figure 2:**
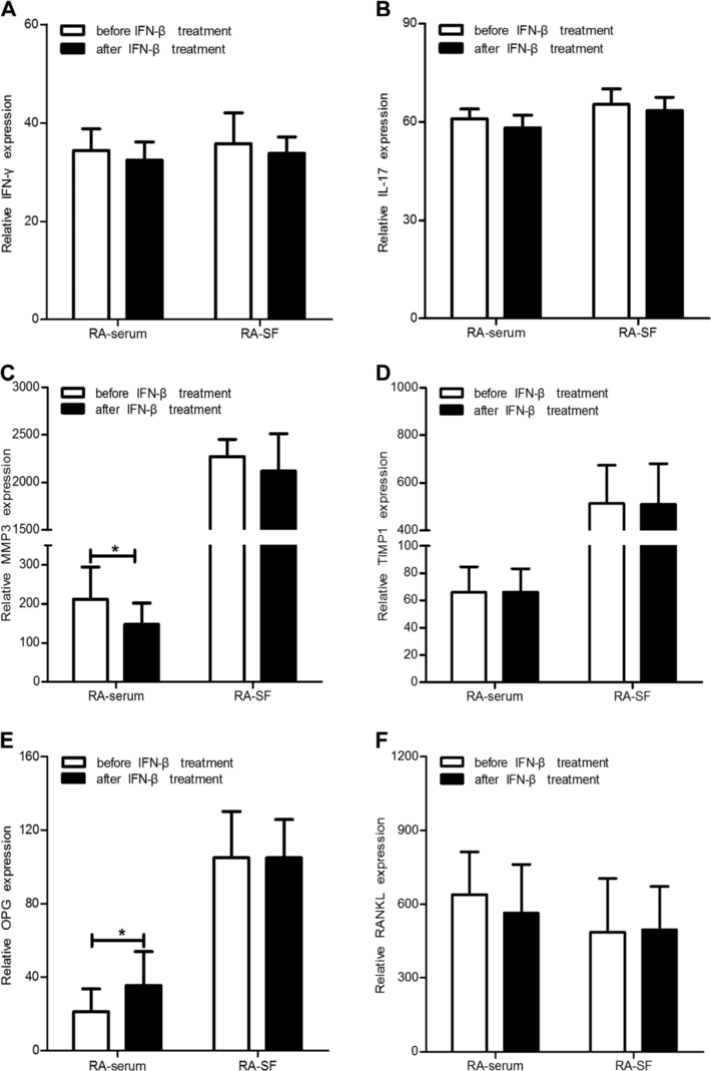
**Cytokine patterns before and after IFN-β treatment in RA serum and SF.** Serum and SF levels of IFN-γ **(A)**, IL-17 **(B)**, MMP-3 **(C),** TIMP-1 **(D),** OPG **(E),** and RANKL **(F)** in RA patients before and after IFN-β administration. *: *P* <0.05.

In this preliminary assessment of exogenous IFN-β intervention in RA patients, we found that the clinical symptoms in some RA patients were partially alleviated, including duration of morning stiffness (min), number of painful joints and swollen joints, and the degree of pain reported by patients. The levels of inflammatory factors (IFN-γ, IL-17, MMP-3, TIMP-1, OPG, and RANKL) in serum and SF were assessed by ELISA both before and one week after treatment with exogenous IFN-β. The levels of IFN-γ and IL-17 appeared to decrease after IFN-β treatment, but there were no significant differences (Figure 2A,B). After IFN-β treatment, the MMP-3 level in serum was decreased (*P* <0.05), but there was no significant change in the levels of MMP-3 in SF or TIMP-1 in either serum or SF (Figure 2C,D). After IFN-β treatment, the OPG level was increased in serum (*P* <0.05), but there were no significant changes in the OPG level in SF or RANKL level in either serum or SF (Figure 2E,F).

### Endogenous IFN-β decreased in CAIA model mice, and exogenous IFN-β may alleviate arthritis severity

**Figure 3:**
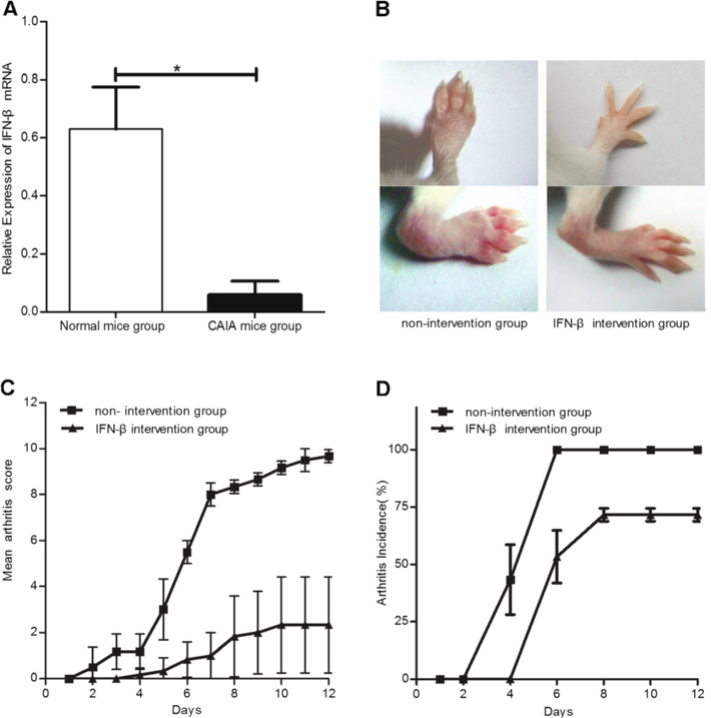
**Endogenous IFN-β expression and the effect of IFN-β treatment on CAIA model mice.** The endogenous expression of IFN-β in the CAIA mice and normal control mice groups **(A)**. Photographs of example hind-paws **(B)**, arthritis scores **(C),** and the morbidity of arthritis **(D)** in the IFN-β intervention and non-intervention groups. *: *P* <0.05.

The CAIA model was successfully induced, and, on Day 12, a lower endogenous IFN-β RNA expression was found in the joints by qRT-PCR (*P* <0.05 vs. normal BALB/c mice) (Figure 3A). After IFN-β administration, the symptoms of the CAIA mice were alleviated and the arthritis scores were decreased compared with the non-intervention group (Figure 3B,C). The incidence of arthritis in the IFN-β intervention group decreased by 30% (Figure 3D).

### Exogenous IFN-β may attenuate bone inflammatory infiltration and cartilage and bone destruction in CAIA model mice

**Figure 4:**
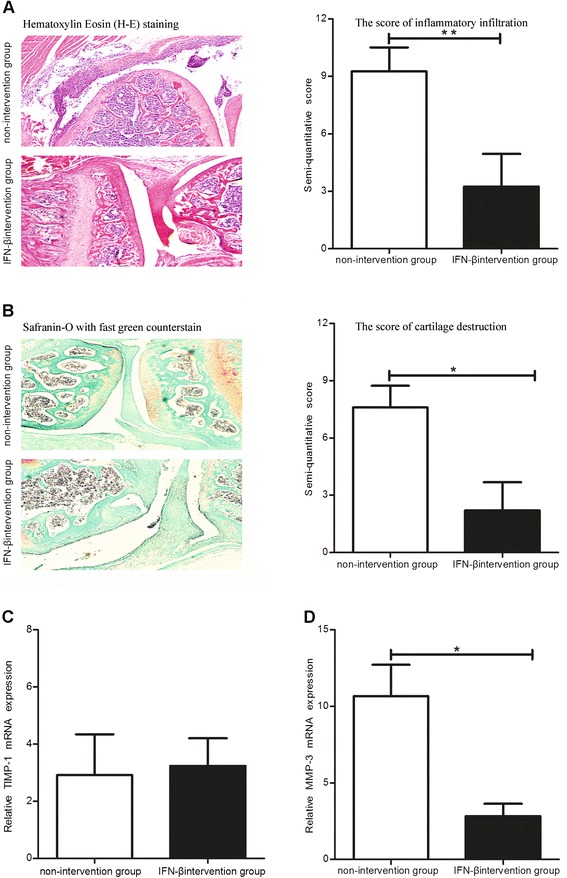
**Effects of exogenous IFN-β treatment on the inflammation and cartilage destruction in CAIA model mice.** The inflammatory cellular infiltration score **(A)**, cartilage injury **(B),** and the levels of MMP-3 **(C)** and TIMP-1 **(D)** in the IFN-β intervention and non-intervention groups *: *P* <0.05.

**Figure 5:**
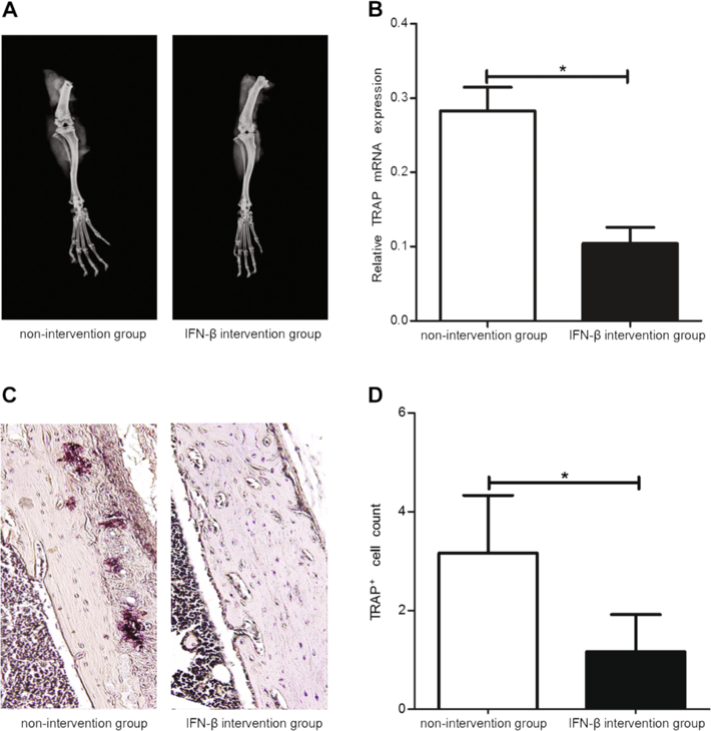
**Effect of exogenous IFN-β administration on the destruction of joint bones.** Ankle joint destruction **(A)**, TRAP mRNA level **(B)**, TRAP staining of joints **(C),** and the number of TRAP-positive multi-nucleated (≥3 nuclei) cells **(D)** in the IFN-β intervention and non-intervention groups. *: *P* <0.05.

The paws and knees of the mice were harvested on Day 12 after injection of the anti-collagen II functional domains antibodies. Compared with the non-intervention group, the HE and safranin-O with fast green counterstain staining revealed a decrease in the number of infiltrated inflammatory cells in the articular cavity (Figure 4A) (*P* <0.01) and an attenuation in amount of cartilage destruction in the IFN-β intervention group (Figure 4B) (*P* <0.05). qRT-PCR was performed to determine the changes in TIMP-1 and MMP-3 expression in the paws of the mice. Although the expression of TIMP-1 mRNA was not changed after IFN-β treatment compared to the non-intervention group (Figure 4C), the expression of MMP-3 mRNA, a mediator of cartilage catabolism, was significantly decreased (Figure 4D) (*P* <0.05). The joint bones of the mice were imaged using molybdenum X-ray to determine the effect of exogenous IFN-β on bone. Compared with the non-intervention group, the bone mineral density was increased (Figure 5A), while the osteoclast marker TRAP mRNA level was decreased in the bones of mouse joints in the IFN-β intervention group (Figure 5B) (*P* <0.05). TRAP staining was also performed to visualize osteoclast infiltration into the bones of mouse joints, and the results showed that the number of osteoclasts was significantly decreased in the IFN-β intervention group (Figure 5C,D) (*P* <0.05).

### RANKL-RANK signaling pathway regulation by exogenous IFN-β in CAIA model mice

**Figure 6:**
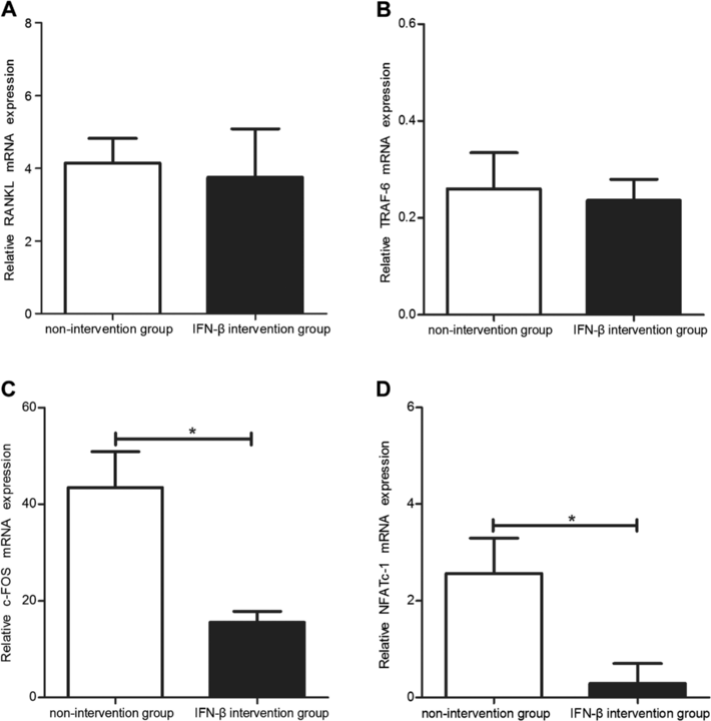
**Changes in the RANKL-RANK signaling pathway after exogenous IFN-β treatment in the CAIA model mice.** The levels of RANKL **(A)**, TRAF6 **(B)**, c-Fos **(C),** and NFATc-1 **(D)** in the joints of mice in the IFN-β intervention and non-intervention groups. *: *P* <0.05.

The expression level of osteoclastogenesis-related RANKL-RANK signaling molecules was detected using qRT-PCR. While there was no change in the expression of upstream molecules RANKL and TRAF-6 (Figure 6A,B), the expression levels of downstream molecules c-Fos and NFATc-1 were significantly decreased in the IFN-β intervention group compared with the non-intervention group (Figure 6C,D) (*P* <0.05).

### RANKL-induced osteoclast differentiation by the RAW264.7 cell line was inhibited by exogenous IFN-β

**Figure 7:**
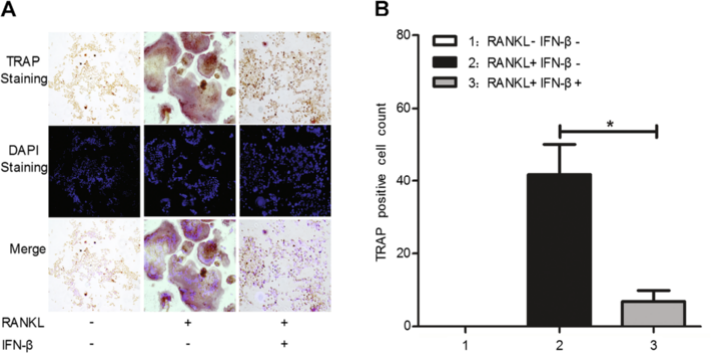
**Effects of exogenous IFN-β administration on RANKL-induced osteoclastogenesis.** TRAP staining **(A)** and the number of TRAP-positive multi-nucleated **(B)** RAW264.7 cells after RANKL and exogenous mouse IFN-β treatments or RANKL treatment alone. *: *P* <0.05.

IFN-β markedly suppressed RANKL-induced osteoclast differentiation in RAW264.7 cells as assessed using TRAP and DAPI staining. Four days after RANKL induction, the number of TRAP-positive osteoclasts was decreased by IFN-β treatment (Figure 7A,B) (*P* <0.05).

## Discussion

To better study RA, it is important to choose a model that accurately reflects the pathology of RA. The CAIA mice model is induced by injecting an anti-collagen antibody cocktail followed by injections of LPS, it offers several key advantages over the classic collagen-induced arthritis (CIA) model, including a rapid disease onset, synchronicity, high uptake rate, and the capacity to use genetically modified mice, such as transgenics and knockouts [18-20]. This model replicates many aspects of the human effector phase of RA [21]. It occurs independently of any direct activity of B and T cells, allowing effector processes to be studied independently of the events that occur during disease induction [22]. The articular inflammation and cellular infiltration characteristics of the effector stage are attributable to deposited immune complexes and activation of complement and Fc receptors (FcR) [21,23]. Cartilage and bone erosion follows the activation of macrophages, lymphocytes, and synoviocytes and production of MMPs and cytokines [21,22].

For the clinical management of RA, a variety of drugs have been used to improve the symptoms, but none of them is effective in all RA patients. For example, although TNF inhibitors have been successful in improving the clinical outcomes for some patients with RA, other patients do not respond to those treatments. The nature and pathogenesis of RA are complicated and likely contribute to the different therapeutic responses. Thus, the treatment of RA is complex and physicians must select an effective therapeutic approach for each patient individually. For example, a prior study suggested that patients with increased basal plasma IFN-β activity respond better to TNF inhibition therapy, while patients with low basal IFN-β levels respond better to anti-B-cell therapy [24].

IFN-β was developed as a therapeutic agent for autoimmune diseases because of its anti-inflammatory activity. Similar to other biological therapies, this treatment is not uniformly effective. In the collagen-induced and adjuvant arthritis animal models, daily systemic administration of IFN-β resulted in a reduction in disease activity and inhibition of cartilage and bone erosion cause by a significant decrease in TNF and IL-6 expression, as well as an increase in IL-10 response at the site of inflammation [8,17]. Clinical trials using IFN-β for treating RA have shown conflicting results [11]. Administration of recombinant IFN-β, in the context of a randomized, double-blind, placebo-controlled clinical trial for the treatment of patients with active RA, however, showed no treatment effect on the clinical or radiographic scores [11]. Consistent with our results, exogenous IFN-β is beneficial for animal models of RA, but the treatment of RA patients with IFN-β has been unsuccessful so far. Thus, the results presented in the present study show the therapeutic use of exogenous IFN-β in RA patients only partly alleviated the disease symptoms. The results in the present study also showed that the expression of endogenous IFN-β in the bones of joints in CAIA model mice was lower than that in normal mice. Therefore, we treated CAIA model mice with exogenous IFN-β beginning at the onset stage, and their arthritis severity was improved, synovial inflammation was attenuated, and destruction of cartilage and bone in the joint were reduced. Unfortunately, we did not measure the expression of endogenous IFN-β in the enrolled RA patients. It is suggested that exogenous IFN-β intervention for RA patients should be used more selectively, and it is possible that exogenous IFN-β might only be useful for RA patients who have low levels of endogenous IFN-β.

The clinical presentation and response to treatment of RA involves many complex immunological and genetic interactions. In addition to its critical antiviral and anti-inflammatory functions, IFN-β also plays an important role in maintaining bone homeostasis, though the exact mechanisms by which exogenous IFN-β reduces RA symptoms, as well as how it maintains bone homeostasis, remain unknown. Accumulating evidence suggests that the bone destruction in RA is mostly caused by osteoclasts [25]. Osteoclasts, derived from monocyte and macrophage lineage precursor cells, are regulated by the receptor activator of nuclear factor-κB (NF-κB) ligand (RANKL) and macrophage colony-stimulating factor (M-CSF). M-CSF promotes osteoclast survival and proliferation, while RANKL is an essential signal for osteoclast differentiation [26]. RANKL exerts its effects by binding to RANK in osteoclasts and their precursors. OPG competes with RANKL as an osteoclast-inhibitor [27]. Thus, the RANKL-RANK signaling pathway is a potential target for preventing joint destruction in RA patients [28]. After binding RANKL, RANK activates c-Fos and tumor necrosis factor-receptor-associated factor 6 (TRAF6), which allows TRAF6 to stimulate the NF-κB and JNK signaling pathways. Interestingly, c-Fos can induce endogenous IFN-β, causing negative feedback regulation of RANKL signaling: IFN-β activates the transcription factor complex interferon-stimulated gene factor-3 (ISGF3), which binds to the interferon-stimulated responsive element (ISRE) on IFN-inducible genes to suppress RANKL-induced c-Fos protein expression [29,30]. We propose that the expression of endogenous IFN-β in some RA patients indicates the activation of an incomplete anti-inflammatory response that may reduce synovial inflammation and, perhaps more importantly, may inhibit bone destruction. Thus, exogenous IFN-β treatment may be a beneficial therapeutic strategy for inhibiting bone degradation in arthritis.

The results of the present study demonstrate for the first time that daily administration of exogenous IFN-β, starting at the onset stage of disease, in the murine CAIA model reduces synovial inflammation and protects against cartilage and bone destruction. Treatment with exogenous IFN-β also resulted in a reduction in osteoclastogenesis, which may be explained by the inhibition of the RANKL-c-Fos signaling pathway activity.

## Conclusions

The marked reduction of arthritic symptoms in CAIA mice, the changes in synovial tissue and joint bones from mice with CAIA after exogenous IFN-β intervention, and the effects of IFN-β on RA patients all support exogenous IFN-β administration as having immunomodulating effects on the CAIA model, and suggest it may reduce joint inflammation and, perhaps more importantly, bone destruction by inhibiting the RANKL-c-Fos signaling pathway activity. Exogenous IFN-β administration should be selectively used in RA patients whose endogenous IFN-β expression is low.

## References

[1] Formica MK, McAlindon TE, Lash TL, Demissie S, Rosenberg L (2010). Validity of self-reported rheumatoid arthritis in a large cohort: results from the Black Women’s Health Study. Arthritis Care Res (Hoboken).

[2] Karlson EW, Chibnik LB, Tworoger SS, Lee IM, Buring JE, Shadick NA, Manson JE, Costenbader KH (2009). Biomarkers of inflammation and development of rheumatoid arthritis in women from two prospective cohort studies. Arthritis Rheum.

[3] Firestein GS (2003). Evolving concepts of rheumatoid arthritis. Nature.

[4] Smolen JS1, Aletaha D, Koeller M, Weisman MH, Emery P (2007). New therapies for treatment of rheumatoid arthritis. Lancet.

[5] Lapadula G, Marchesoni A, Armuzzi A, Blandizzi C, Caporali R, Chimenti S, Cimaz R, Cimino L, Gionchetti P, Girolomoni G, Lionetti P, Marcellusi A, Mennini FS, Salvarani C (2014). Adalimumab in the treatment of immune-mediated diseases. Int J Immunopathol Pharmacol.

[6] Loma I, Heyman R (2011). Multiple sclerosis: pathogenesis and treatment. Curr Neuropharmacol.

[7] Kremenchutzky M, Morrow S, Rush C (2007). The safety and efficacy of IFN-beta products for the treatment of multiple sclerosis. Expert Opin Drug Saf.

[8] Adriaansen J, Kuhlman RR, van Holten J, Kaynor C, Vervoordeldonk MJ, Tak PP (2006). Intraarticular interferon-beta gene therapy ameliorates adjuvant arthritis in rats. Hum Gene Ther.

[9] van Holten J, Plater-Zyberk C, Tak PP (2002). Interferon-β for treatment of rheumatoid arthritis?. Arthritis Res.

[10] van Holten J, Smeets TJ, Blankert P, Tak PP (2005). Expression of interferon beta in synovial tissue from patients with rheumatoid arthritis: comparison with patients with osteoarthritis and reactive arthritis. Ann Rheum Dis.

[11] van Holten J, Pavelka K, Vencovsky J, Stahl H, Rozman B, Genovese M, Kivitz AJ, Alvaro J, Nuki G, Furst DE, Herrero-Beaumont G, McInnes IB, Musikic P, Tak PP (2005). A multicentre, randomised, double blind, placebo controlled phase II study of subcutaneous interferon beta-1a in the treatment of patients with active rheumatoid arthritis. Ann Rheum Dis.

[12] Smeets TJ, Dayer JM, Kraan MC, Versendaal J, Chicheportiche R, Breedveld FC, Tak PP (2000). The effects of interferon-β treatment of synovial inflammation and expression of metalloproteinases in patients with rheumatoid arthritis. Arthritis Rheum.

[13] Arnett FC, Edworthy SM, Bloch DA, McShane DJ, Fries JF, Cooper NS, Healey LA, Kaplan SR, Liang MH, Luthra HS, Medsger TA, Mitchell DM, Neustadt DH, Pinals RS, Schaller JG, Sharp JT, Wilder RL, Hunder GG (1988). The American Rheumatism. Association 1987 Revisedcriteria for the classification of rheumatoid arthritis. Arthritis Rheum.

[14] Kellgren JH, Lawrence JS (1957). Radiological assessment of osteo-arthrosis. Ann Rheum Dis.

[15] Seeuws S1, Jacques P, Van Praet J, Drennan M, Coudenys J, Decruy T, Deschepper E, Lepescheux L, Pujuguet P, Oste L, Vandeghinste N, Brys R, Verbruggen G, Elewaut D (2010). A multiparameter approach to monitor disease activity in collagen-induced arthritis. Arthritis Res Ther.

[16] Al-Dujaili SA, Lau E, Al-Dujaili H, Tsang K, Guenther A, You L (2011). Apoptotic osteocytes regulate osteoclast precursor recruitment and differentiation in vitro. J Cell Biochem.

[17] van Holten J, Reedquist K, Sattonet-Roche P, Smeets TJ, Plater-Zyberk C, Vervoordeldonk MJ, Tak PP (2004). Treatment with recombinant interferon-beta reduces inflammation and slows cartilage destruction in the collagen-induced arthritis model of rheumatoid arthritis. Arthritis Res Ther.

[18] Hutamekalin P, Saito T, Yamaki K, Mizutani N, Brand DD, Waritani T, Terato K, Yoshino S (2009). Collagen antibody-induced arthritis in mice: development of a new arthritogenic 5-clone cocktail of monoclonal anti-type II collagen antibodies. J I Methods.

[19] Khachigian LM (2006). Collagen antibody-induced arthritis. Nat Protoc.

[20] Dimitrova P, Ivanovska N, Belenska L, Milanova V, Schwaeble W, Stover C (2012). Abrogated RANKL expression in properdin-deficient mice is associated with better outcome from collagen-antibody-induced arthritis. Arthritis Res Ther.

[21] Nandakumar KS, Holmdahl R (2006). Antibody-induced arthritis: disease mechanisms and genes involved at the effector phase of arthritis. Arthritis Res Ther.

[22] Croxford AM, Whittingham S, McNaughton D, Nandakumar KS, Holmdahl R, Rowley MJ (2013). Type II collagen-specific antibodies induce cartilage damage in mice independent of inflammation. Arthritis Rheum.

[23] Bender AT, Spyvee M, Satoh T, Gershman B, Teceno T, Burgess L, Kumar V, Wu Y, Yang H, Ding Y, Akare S, Chen Q (2013). Evaluation of a candidate anti-arthritic drug using the mouse collagen antibody induced arthritis model and clinically relevant biomarkers. Am J Transl Res.

[24] Thurlings RM, Boumans MJH, Tekstra J, Bijlsma JWJ, Van Baarsen LGM, Vos K, Bos C, Kirou KA, Crow MK, Verweij CL, Tak PP (2009). The type I IFN signature is a negative predictor of the clinical response to rituximab treatment in RA. Arthritis Rheum.

[25] Søe K, Merrild DM, Delaissé JM (2013). Steering the osteoclast through the demineralization-collagenolysis balance. Bone.

[26] Xing L, Schwarz EM, Boyce BF (2005). Osteoclast precursors, RANKL/RANK, and immunology. Immunol Rev.

[27] Martin TJ (2013). Historically significant events in the discovery of RANK/RANKL/OPG. World J Orthop.

[28] Vis M, Güler-Yüksel M, Lems WF (2013). Can bone loss in rheumatoid arthritis be prevented?. Osteoporos Int.

[29] Mohamed SG, Sugiyama E, Shinoda K, Taki H, Hounoki H, Abdel-Aziz HO, Maruyama M, Kobayashi M, Ogawa H, Miyahara T (2007). Interleukin-10 inhibits RANKL-mediated expression of NFATc1 in part via suppression of c-Fos and c-Jun in RAW264.7 cells and mouse bone marrow cells. Bone.

[30] Takayanagi H, Kim S, Matsuo K, Suzuki H, Suzuki T, Sato K, Yokochi T, Oda H, Nakamura K, Ida N, Wagner EF, Taniguchi T (2002). RANKL maintains bone homeostasis through c-Fos-dependent induction of interferon-beta. Nature.

